# A methodology for estimating SARS-CoV-2 importation risk by air travel into Canada between July and November 2021

**DOI:** 10.1186/s12889-024-18563-1

**Published:** 2024-04-19

**Authors:** Rachael M. Milwid, Vanessa Gabriele-Rivet, Nicholas H. Ogden, Patricia Turgeon, Aamir Fazil, David London, Simon de Montigny, Erin E. Rees

**Affiliations:** 1 Public Health Risk Sciences Division, National Microbiology Laboratory, Public Health Agency of Canada, St-Hyacinthe, QC Canada; 2 Public Health Risk Sciences Division, National Microbiology Laboratory, Public Health Agency of Canada, Guelph, Guelph, ON Canada; 3 Department of Pathology and Microbiology, Faculty of Veterinary Medicine, Université de Montréal, Saint-Hyacinthe, QC Canada; 4 Physique Des Particules, Université de Montréal, Faculté Des Arts Et Des Sciences, Montréal, QC Canada; 5 Emergency Management Branch, Global Public Health Intelligence Network Tiger Team, Public Health Agency of Canada, Ottawa, ON Canada; 6 Epidemiology of Zoonoses and Public Health Research Unit, Faculté de médecine vétérinaire, Université de Montréal, Saint-Hyacinthe, QC Canada

**Keywords:** COVID-19, Importation risk, Air travel, Mathematical model, Pre-departure molecular testing, Canada

## Abstract

**Background:**

Estimating rates of disease importation by travellers is a key activity to assess both the risk to a country from an infectious disease emerging elsewhere in the world and the effectiveness of border measures. We describe a model used to estimate the number of travellers infected with SARS-CoV-2 into Canadian airports in 2021, and assess the impact of pre-departure testing requirements on importation risk.

**Methods:**

A mathematical model estimated the number of essential and non-essential air travellers infected with SARS-CoV-2, with the latter requiring a negative pre-departure test result. The number of travellers arriving infected (i.e. imported cases) depended on air travel volumes, SARS-CoV-2 exposure risk in the departure country, prior infection or vaccine acquired immunity, and, for non-essential travellers, screening from pre-departure molecular testing. Importation risk was estimated weekly from July to November 2021 as the number of imported cases and percent positivity (PP; i.e. imported cases normalised by travel volume). The impact of pre-departure testing was assessed by comparing three scenarios: baseline (pre-departure testing of all non-essential travellers; most probable importation risk given the pre-departure testing requirements), counterfactual scenario 1 (no pre-departure testing of fully vaccinated non-essential travellers), and counterfactual scenario 2 (no pre-departure testing of non-essential travellers).

**Results:**

In the baseline scenario, weekly imported cases and PP varied over time, ranging from 145 to 539 cases and 0.15 to 0.28%, respectively. Most cases arrived from the USA, Mexico, the United Kingdom, and France. While modelling suggested that essential travellers had a higher weekly PP (0.37 – 0.65%) than non-essential travellers (0.12 – 0.24%), they contributed fewer weekly cases (62 – 154) than non-essential travellers (84 – 398 per week) given their lower travel volume. Pre-departure testing was estimated to reduce imported cases by one third (counterfactual scenario 1) to one half (counterfactual scenario 2).

**Conclusions:**

The model results highlighted the weekly variation in importation by traveller group (e.g., reason for travel and country of departure) and enabled a framework for measuring the impact of pre-departure testing requirements. Quantifying the contributors of importation risk through mathematical simulation can support the design of appropriate public health policy on border measures.

**Supplementary Information:**

The online version contains supplementary material available at 10.1186/s12889-024-18563-1.

## Background

Government public health organisations are responsible for assessing the risk of importation of infectious diseases (e.g. [1]). To be effective, such risk assessments can use modelling methods that integrate data on incoming travel volumes from source endemic/epidemic locations through the global travel network, and country-specific epidemiological and vaccine coverage data [2, 3]. In addition to assessing the spatio-temporal risk of importation, models can also be used to quantify the effectiveness of specific prevention strategies prior to their implementation, or post-hoc as a means of on-going evaluation and support for preparedness [4]. This can be accomplished by comparing estimated importation rates with measures in place against scenarios in which border measures are removed.

**Fig. 1:**
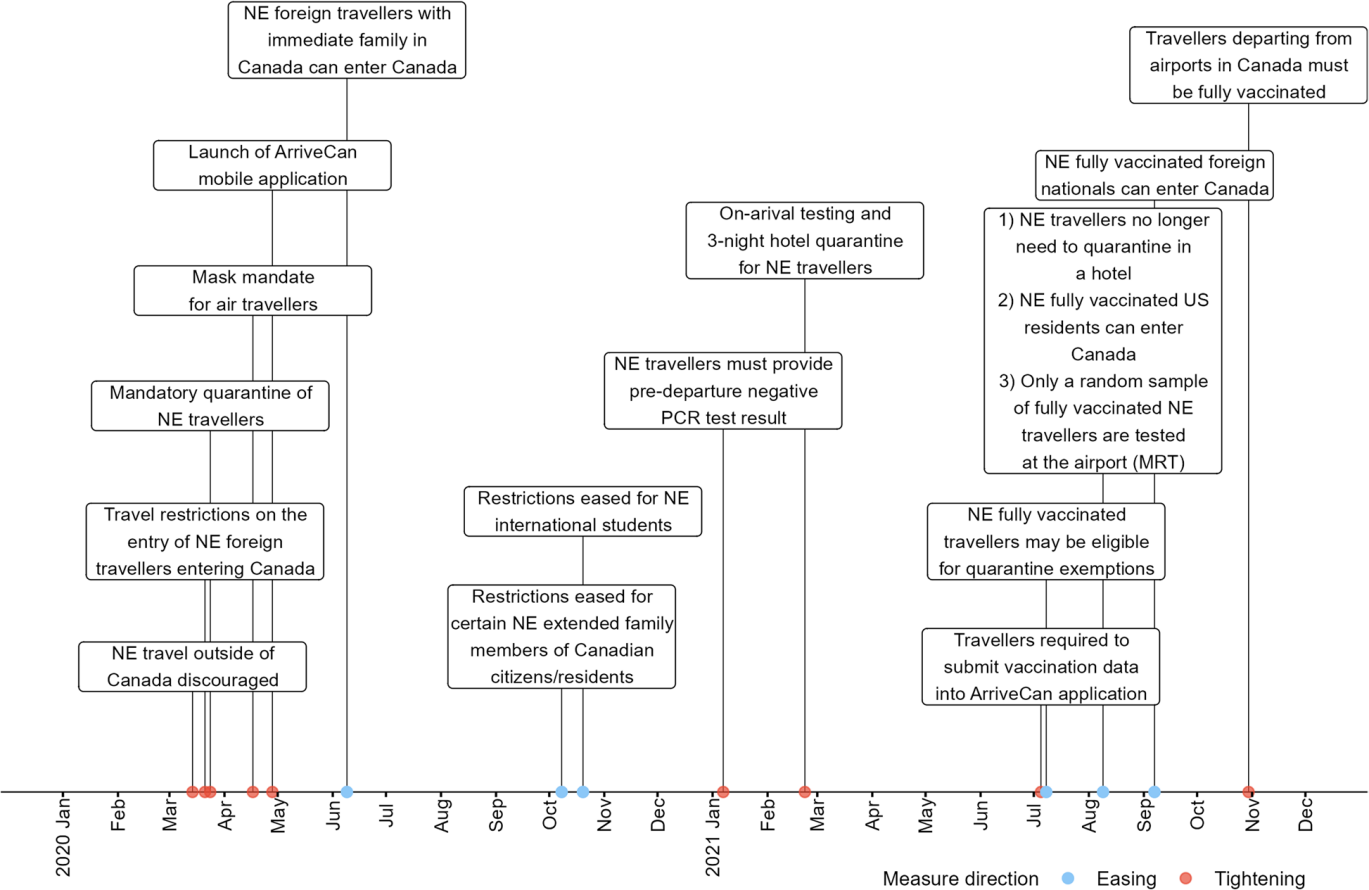
Summary of Canadian border measures implemented and eased in 2020–2021 [6, 13–15]. NE = Non-essential

SARS‑CoV‑2, the causative agent of COVID-19, spread rapidly across the world resulting in nearly 300 million reported cases and 5.5 million reported deaths by the end of 2021 [5]. From March 2020 to September 2022, the Canadian government implemented border measures to slow the importation of COVID-19 cases arising from international air travel [6] (Fig. 1). These measures included restrictions on foreign nationals entering Canada [6], flight suspensions from selected countries [7], vaccination requirements to enter Canada [8], pre-departure molecular testing for SARS-CoV-2 within 72 h of departure [9], quarantine and further testing upon entry into Canada [10, 11], and post-entry testing. Some travellers were exempt from some or all of the border measures depending on their reason for travel (e.g. providing an essential service) [12].

During the COVID-19 pandemic, importation models were used to estimate the number of imported cases from domestic and international travel, and assess the impact of border measures [16–19]. In Canada, mathematical models were developed within the first few months of the pandemic to assess the impact of importation on local COVID-19 transmission in specific provinces (e.g. Québec and Ontario [19], and Newfoundland and Labrador [20]). At the national-level, an importation modelling method was implemented by the Public Health Agency of Canada’s (PHAC) modelling team to assess possible rates of importation of cases throughout the pandemic, with and without border measures. This study aimed to describe the mathematical model developed by PHAC and estimate the weekly importation risk from air travellers into Canadian airports from July to November 2021 as measured by the number of travellers infected with SARS-CoV-2 (i.e. imported cases) and percent positivity, PP (i.e. imported cases normalised by total travel volume). In addition, the impact of pre-departure testing of non-essential travellers to reduce importation risk was assessed by comparing estimated imported cases against counterfactual scenarios.

## Methods

The model operates at a daily time step to estimate the weekly number of air travellers arriving infected with SARS-CoV-2 at the airport-level from July to November 2021. The model was adapted from a mathematical model previously used to estimate importation risk of dengue and COVID-19 [2, 18]. The key model adaptations adjusted for underreporting in COVID-19 case counts, accounted for the impacts of vaccination and pre-departure testing for SARS-CoV-2 to reduce importation risk, and stratified importation risk by SARS-CoV-2 variants of concern (VOC) and variants of interest (VOI).

### Air travel volume data

Model input for air travel volumes was derived from two data sources. Daily travel volumes from each country of departure (i.e. the country from which travel to Canada was initiated) to Canada were derived using Canada Border Services Agency’s (CBSA) Advanced Passenger Information in combination with the overall passage data from CBSA (Additional File 1). Monthly travel volumes for each itinerary from the origin airport to the final Canadian destination airport were obtained from the International Air Transport Authority (IATA) [21]. Finally, the CBSA travel volumes were distributed in proportion to the IATA travel volumes to derive model input at the daily and airport levels.

### Traveller groups

In the model, travellers were stratified as essential or non-essential based on their reason for travel. Non-essential travellers, which included those who travelled for personal reasons (e.g. tourism, education), were assumed to have a negative pre-departure molecular test result three days prior to their scheduled departure [11], while essential travellers were exempt from that requirement. Between November 2020 and October 2022, non-essential travellers were required to submit COVID-19 related information [22, 23] via the Government of Canada’s (GoC) digital ArriveCan platform at each entry into Canada. This data source, in combination with the CBSA ContactTrace program, were used to derive the weekly country-specific proportions of non-essential travellers in the model ([24]; Additional file 1).

Travellers were also characterized as being Canadian or foreign residents to distinguish their place of residence as being in Canada or another country, respectively. In the model, Canadian residents were assumed to have spent all their time in Canada, except for the period in which they travelled to a non-Canadian country where they could become infected with COVID-19 and then import the infection into Canada. This time spent outside of Canada was assumed to follow a normal distribution with a mean of 15 days and a standard deviation of 2 days according to recent estimates [25]. Foreign residents were assumed to reside and spend their time only in the country of departure before travel to Canada. This was the country in which they could be infected with SARS-CoV-2 prior to entering Canada. Model input for the country-specific weekly proportions of Canadian and foreign residents were derived from CBSA’s Advanced Passenger Information data (for essential travellers) and ArriveCan and ContactTrace data (for non-essential travellers, Additional file 1).

Finally, travellers were stratified by vaccination status to account for any vaccine-induced immunity. For non-essential travellers, the weekly country-specific distributions of vaccine statuses were derived from the ArriveCan and ContactTrace data and could be one of: unvaccinated, partially vaccinated with a GoC approved vaccine, partially vaccinated with a non-GoC approved vaccine, fully vaccinated with GoC approved vaccines, fully vaccinated with non-GoC approved vaccines or fully vaccinated with a mixture of GoC approved and non-GoC approved vaccines. Hereafter, *partially vaccinated* refers to vaccination with one dose of a two dose vaccine regime while *fully vaccinated* refers to one dose of a one dose vaccine regime or two doses of a two dose vaccine regime. The vaccination status of essential travellers was not available from the ArriveCan data because these travellers were not required to provide proof of vaccination during the study period. Model input for the daily distributions of vaccination statuses in essential travellers were assumed to follow the vaccine coverage for the country of departure (foreign resident travellers) or for Canada (Canadian resident travellers) as reported by Our World in Data (OWD; [5]). Vaccination status for essential travellers in the model included only unvaccinated, partially vaccinated or fully vaccinated because OWD did not provide information on vaccine type for us to distinguish between GoC approved or otherwise.

### Correcting for underreporting of COVID-19 cases

Reported COVID-19 case data were likely underestimated due to asymptomatic transmission, incomplete testing and imperfect test sensitivity and reporting systems [26]. We derived country-specific correction factors to inflate case data and better reflect the true prevalence (Additional File 1). A semi-Bayesian probabilistic bias approach was used to estimate the number of true cases at the country level, using reported case data and testing rates [27]. We adapted the method to also account for the evolving population-level immunity due to previous COVID-19 infections and increasing vaccination rates. True case counts were estimated from March to August 2020 and then monthly thereafter to reduce instability in estimates caused by sparse case data at the onset of the pandemic and low testing rates [27]. The estimated true case count was divided by the reported case count [5, 28, 29] in order to obtain country-specific correction factors for each time period from March 2020 onwards. Finally, a regression modelling approach was implemented using the country-level Gross National Income (GNI) as a predictor [30] and the calculated correction factor as the dependent variable. This regression model was used to impute the missing correction factors for countries that did not have case, testing, or vaccination data. The GNI was used as a proxy for the effectiveness of the country surveillance system to detect, test and report COVID-19 cases [30].

### Model formulation

The probability of a traveller arriving in Canada infected with SARS-CoV-2 accounts for the vaccination status of the traveller and potential immunity acquired from a previous infection in their country of residence (*cr*). For simplicity, it was assumed that infection- and vaccine-induced immunity did not wane from the beginning of the pandemic until the end of the study period, and prior infections provided complete immunity against re-infection. The probability of a traveller having infection-acquired immunity on any given day *d* and in country of residence *cr* $({Pinf}_{cr,d})$ was calculated as the cumulating proportion of residents reported to have had COVID-19 given the 2020 country population size [5, 31, 32]. For an essential traveller, the probability of vaccine-acquired protection $(Pvac{c\_E}_{cr,d})$ on any given day *d* and in country of residence *cr*, was equal to:

1 $$Pvac{c\_E}_{cr,d}={\sum }_{status} Pro{p}_{cr,d, status}\times {VE}_{cr,status}$$

where ${VE}_{cr,status}$, vaccine effectiveness, is the probability that a traveller had complete immunity against infection which varied according to COVID-19 vaccination status (partially or fully vaccinated) and the *cr* for the assumed type of vaccine (mRNA vaccines or others) (Additional file 1: Table A2); and $Pro{p}_{cr,d, status}$ represents the proportion of the population in country $cr$ for each vaccination status on day *d*. Since vaccination status information was available for non-essential travellers, their probability of vaccine-acquired protection $(Pvac{c\_NE}_{cr,status})$ was equal to the associated vaccine effectiveness ${VE}_{cr,status}$.

The probability of a traveller arriving in Canada infected with SARS-CoV-2 depended on their risk of exposure in the country of departure, *cd*, prior to departure for Canada. The daily probability of infection $({\beta }_{cd,d})$ for a susceptible person on a given day *d* in country *cd* was calculated as the number of new cases (corrected for underreporting) out of the total susceptible population (i.e. the proportion of the population that was not immune to infection with COVID-19 due to prior infection or vaccination). Based on this daily probability of infection, the probability of a traveller arriving in Canada infected with SARS-CoV-2 was calculated according to the traveller’s reason for travel (i.e. essential or non-essential). For an essential traveller, the probability of importation, (${P\_E}_{s,cd,cr};$ Eq. 2 and Additional file 1), on travel day *s* was based on the traveller’s probability of acquiring infection on any of the *n* days prior to departure to Canada, given that they did not have infection-acquired protection $\left(1-{Pinf}_{cr,d}\right)$ or vaccine-acquired protection $\left(1-{Pvacc\_E}_{cr,d}\right)$. Here *n* represents the sum of the latent and infectious periods for SARS-CoV-2 infections (Table 1). The probability of importation for a non-essential traveller, (${P\_NE}_{s,cd,cr, status}$; Eq. 3 and Additional file 1), was based on the traveller’s probability of acquiring infection on any of the (*n -*$\mu$*)* days prior to the test day and receiving a false negative test result on test day, or not being infected on test day and acquiring infection after completing the test prior to departure. Here $\mu$ represents the number of days between the test and travel days (i.e. set at three days in the model). An estimated molecular test sensitivity (*se*) of 60% was implemented, which represented the mean value when accounting for the variation in sensitivity with respect to time since infection ([33, 34]; Additional file 1). Similar to essential travellers, the probability of importation for non-essential travellers is conditional on not having infection-acquired protection $\left(1-{Pinf}_{cr,d}\right)$ or vaccine-acquired protection $\left(1-{Pvacc\_NE}_{cr,status}\right)$.

2 $${P\_E}_{s,cd,cr}=\left[x -\prod_{d={s-i} }^{s-1}\left(1-{\beta }_{cd,d}\right)\right]\left(1-{Pinf}_{cr,s-\left(i+1\right)}\right)\left(1-{Pvacc\_E}_{cr, s-\left(i+1\right)}\right)$$

3 $${P\_NE}_{s,cd,cr, status}=\left[\left(1-se\right)x+se\prod_{d={s-i} }^{s-\left(\mu +1\right)}\left(1-{\beta }_{cd,d}\right)-\prod_{d={s-i} }^{s-1}\left(1-{\beta }_{cd,d}\right)\right]\left(1-{Pinf}_{cr,s-\left(i+1\right)}\right)\left(1-{Pvacc\_NE}_{cr,status}\right)$$

where

$$i=\left\{\begin{array}{c}t_c,\;when\;traveller\;is\;a\;Canadian\;resident\\n,\;when\;traveller\;is\;aforeign\;resident\end{array}\right.$$

$$x=\left\{\begin{array}{c}\prod_{d=s-t_c}^{s-(n+1)}(1-\beta_{cd,d}),\;when\;traveller\;is\;a\;Canadian\;resident\;and\;t_c>n\\1,\;when\;traveller\;is\;a\;Canadian\;resident\;and\;t_c\leq\;n\;or\;a\;foreign\;traveller\end{array}\right.$$

where ${t}_{c}$ is the number of days spent in the country of departure $cd$ prior to leaving for Canada. For foreign residents, it was assumed that ${{\text{t}}}_{{\text{c}}}>{\text{n}}$.

**Table 1:** Parameter values used in a COVID-19 importation risk model to Canada

Parameters	Symbol	Distribution	Values	References
Time spent in non-Canadian country (days) for Canadian travellers	${t}_{c}$	Νormal (mean, SD)	mean = 15, SD = 2	[25]
Time spent in non-Canadian country (days) for Foreign travellers		NA	Time since beginning of pandemic to date of travel to Canada	Assumed
Latent period (days)	NA	Νormal (mean, SD)	mean = 3.5, SD = 1	[35–37]
Infectious period (days)	NA	Νormal (mean, SD)	mean = 12, SD = 4	[35–37]
Time between pre-departure molecular test and travel to Canada (days)	$\upmu$	Fixed value	3	Assumed
Sensitivity (%)	$Se$	Fixed value	60	[38]
Vaccine effectiveness	$Ve$	Νormal (mean, SD)	mean = See Table A2 SD = 0.015	See Table A2

Finally, the total number of importations (${I}_{w}$) for every epi-week, *w*, was calculated using the probability of air travellers arriving infected (${P}_{k,\upgamma ,s}$) for each airport-level origin–destination travel route (*k*), each travel group (γ, i.e. Canadian or foreign resident, vaccination status, essential or non-essential traveller) and each day of the week ($s$), and the corresponding travel volume (${v}_{k,\upgamma ,s}$):

4 $${I}_{w}=\sum_{k,\upgamma ,\mathrm{ s} }\left[{P}_{k,\upgamma ,s}\times {v}_{k,\upgamma ,s}\right]$$

Importation estimates were stratified by VOCs and VOIs listed by the USA Centers for Disease Control and Prevention. It was assumed that the proportion of variants reported in the GISAID database [39] for each country during a three-week period (including the week modelled and the two prior weeks) was the same proportion that would be observed in infected travellers arriving in Canada from these countries.

### Modelling importation risk and counterfactual scenarios

We used the model to estimate importation risk from July 11 to November 27, 2021 under the assumption that all non-essential travellers were required to have a negative molecular pre-departure test result three days prior to departure for Canada. As well as being our most probable estimate of the true importation risk given the testing requirements that were in effect during the modelled time period, these model estimates formed our baseline to compare with two counterfactual scenarios. Model output is presented by country of departure, SARS-CoV-2 variant and traveller groups. In addition, the number of infected travellers arriving at each of Canada’s four largest airports (Toronto Pearson, Montréal-Trudeau, Vancouver International, and Calgary International) as their final destination are presented. Finally, we mapped country-level model outputs in terms of the cumulative number of importations, percent positivity, and travel volumes for the total study period using ArcGIS Pro version 2.9.0 (ESRI, Redlands, CA).

Two counterfactual scenarios were simulated from July 11 to November 27, 2021 to measure the impact of pre-departure testing on non-essential travellers to reduce importation risk as compared to the baseline. For counterfactual scenario 1, fully vaccinated (with or without GoC approved vaccines) non-essential travellers were not tested, and for counterfactual scenario 2 there was no testing of any non-essential travellers. For both counterfactual scenarios, the model was run for all non-essential travellers, whereas outputs from the baseline scenario were used for essential travellers. The weekly percent change in the total number of imported cases for each counterfactual scenario was compared to the baseline scenario.

Model stochasticity was implemented through the distributions of parameter input values for vaccine effectiveness, latent and infectious periods, and for Canadian travellers, travel duration. For each of these parameters, a value was randomly chosen from a pre-defined distribution (Table 1) for every category of traveller, with these categories consisting of unique combinations of origin–destination airport pathway, essential status and day. The baseline and counterfactual scenarios were simulated 50 times. We only present the mean results because the confidence intervals were too narrow to visualise in the plots. All model simulations and analyses were conducted in R version 4.1.0 [40].

## Results

**Fig. 2:**
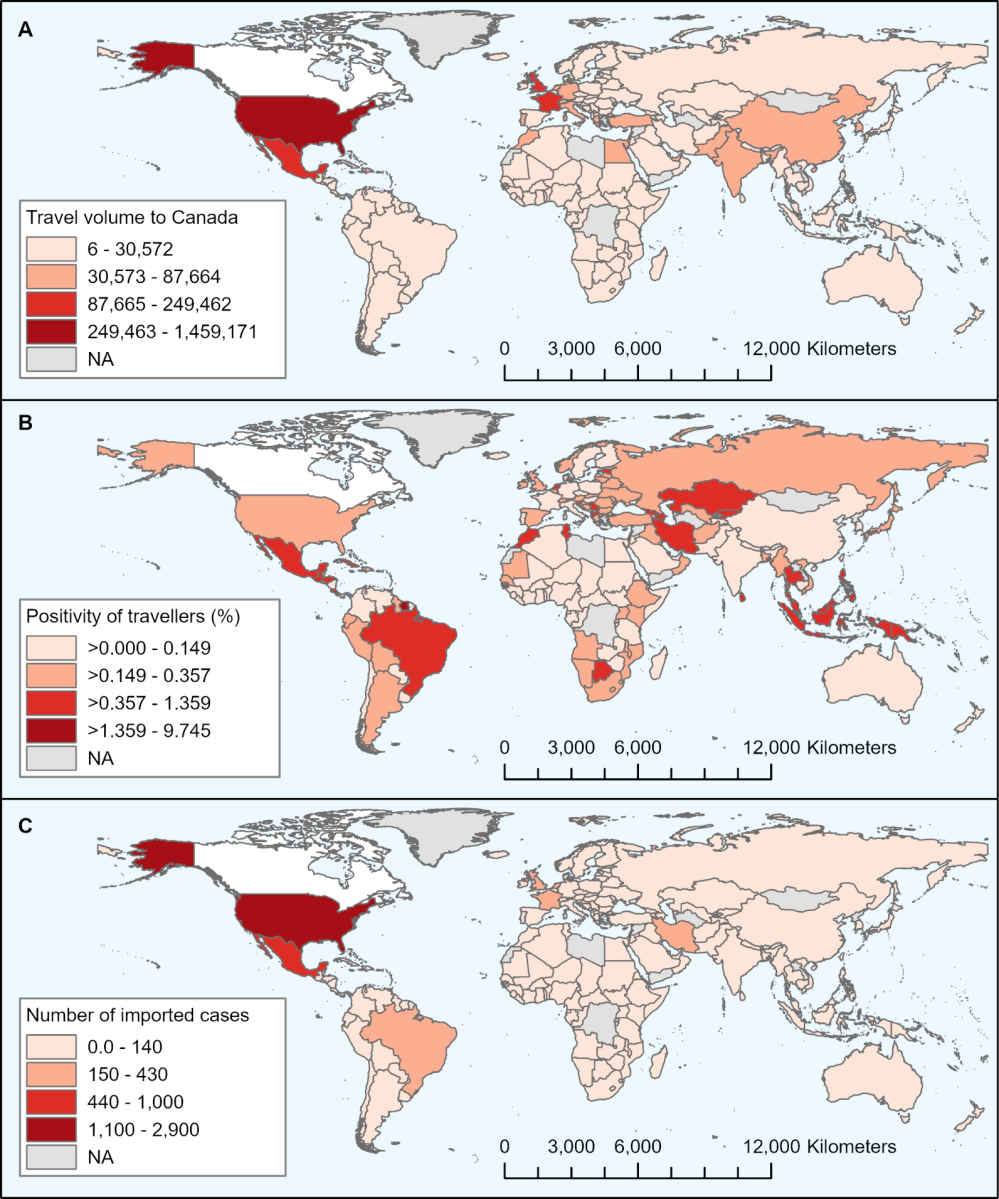
Maps illustrating results at the country of departure level from July 11, 2021 to November 27, 2021 for **A** estimated travel volume to Canada, and model estimates for **B** COVID-19 percent positivity of travellers entering Canada, and **C** number of imported COVID-19 cases to Canada. The destination country, Canada, is shown in white. Countries in grey either have unavailable travel volume data and/or reported case counts

**Fig. 3:**
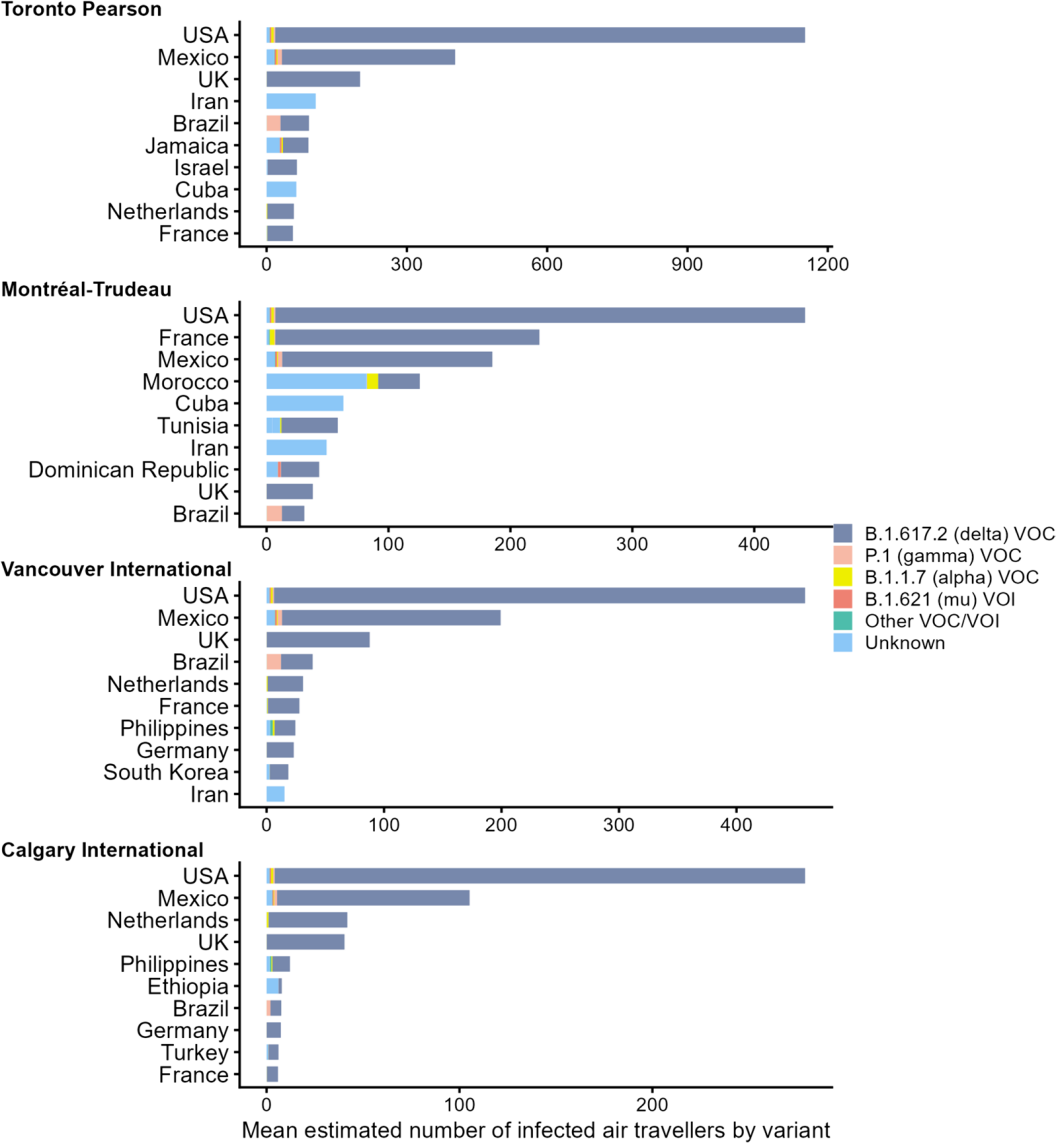
Model output for the mean number of SARS-CoV-2 infected air travellers by variant and country of departure arriving at their final destination in one of the four largest Canadian airports, as estimated from July 11 to November 27, 2021

The importation model estimated that a total of 7,863 infected travellers entered Canada by air from July 11 to November 27, 2021. Most cases originated from the USA (2,890 cases), the country with the highest incoming travel volume to Canada (1.46 million travellers) and a PP of 0.198% (Fig. 2a, b). Other countries with a high risk of importation were Mexico (1,034 cases; 0.414% PP; 249,462 travellers), the United Kingdom (429 cases; 0.277% PP; 154,715 travellers), and France (335 cases, 0.145% PP; 230,295 travellers) (Fig. 2). The relative ranking of contributing countries evolved over time, and differed between destination airports (Figs. 2 and 3, and Fig. A2 in Additional file 1).

The composition of SARS-CoV-2 variants also varied between airports and through time. Throughout the study period the Delta variant was modelled to be the predominant infectious agent in travellers arriving at the Canadian destination airports. There were also estimated contributions from the Gamma, Mu, and Alpha variants, especially prior to August (Fig. 3; Fig. A2 in Additional file 1).

**Fig. 4:**
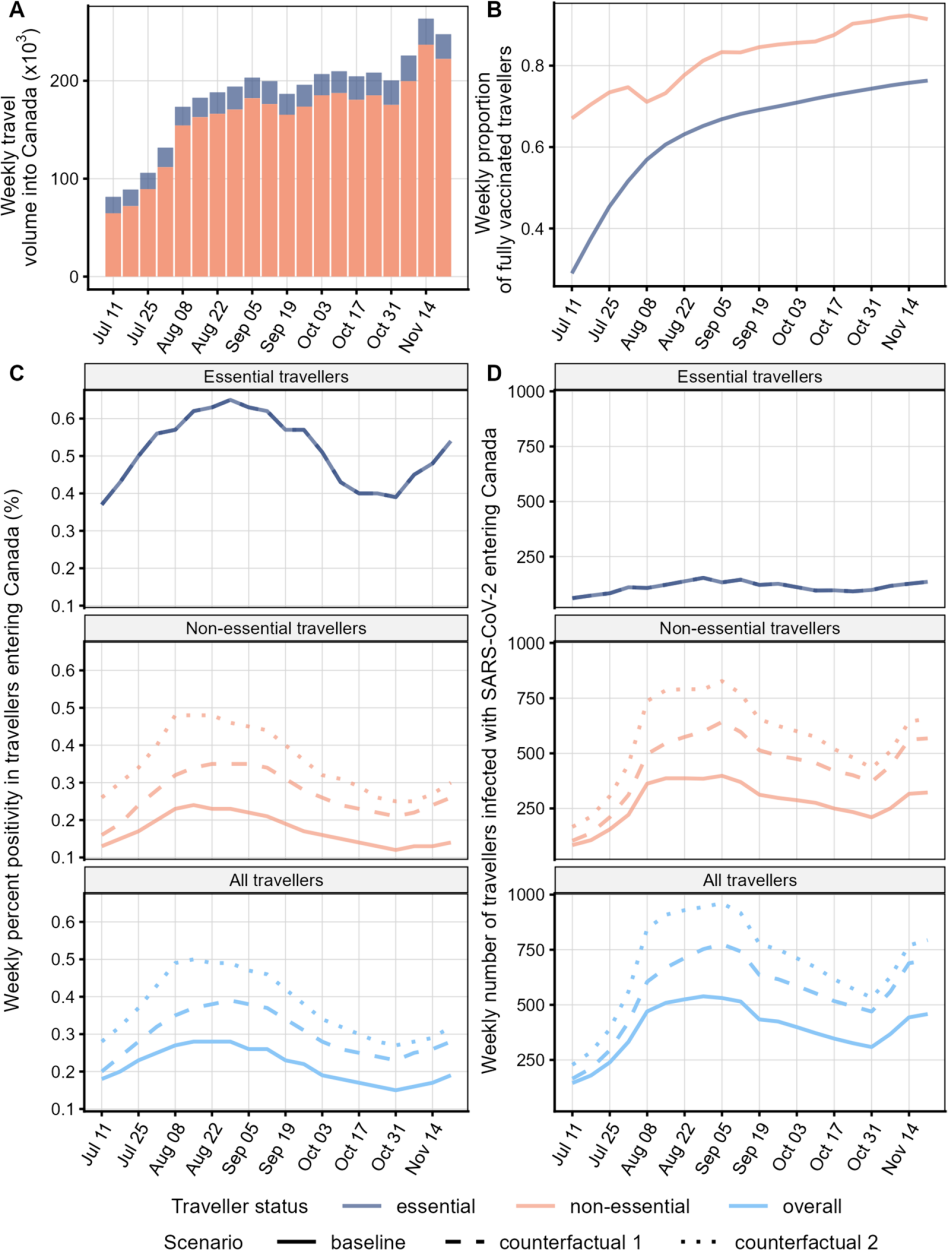
Weekly model inputs for the study period (July to November 2021) for **A** estimated travel volumes into Canada for essential and non-essential travellers, **B** proportions of fully vaccinated travellers estimated for essential travellers given global vaccine coverage [5] and reported for non-essential travellers [23], and model output for **C** percent positivity and **D** number of imported COVID-19 cases into Canada as stratified into essential and non-essential travellers and combined (overall) for the baseline scenario (pre-departure testing of all non-essential travellers), counterfactual scenario 1 (no pre-departure testing of fully vaccinated non-essential travellers) and counterfactual scenario 2 (no pre-departure testing of any non-essential travellers). In **C**) and **D**), the essential traveller curve is identical for all three scenarios since the model for essential travellers was not repeated for the counterfactual scenarios

Output from the importation model suggests that the number of imported cases and PP also varied over time. There was a peak in August, followed by a decrease until the end of October, and a subsequent increase in November (Fig. 4). In the baseline scenario, the mean weekly number of imported cases ranged from 145 to 539 cases and PP ranged from 0.15 to 0.28%. Most cases were imported by non-essential travellers (range: 84–398 per week), who comprised the largest proportion of travel volume (range: 79–90% per week) and populations with full vaccination status (range: 67–92% per week). In contrast, essential travellers had fewer imported cases (range: 62–154 per week), with a smaller travel volume (range: 10–21% per week) and populations of full vaccination status (range: 29–76% per week). Despite having lower importation numbers, the PP in essential travellers was consistently higher (range: 0.37–0.65% per week) than non-essential travellers (range: 0.12–0.24% per week).

**Fig. 5:**
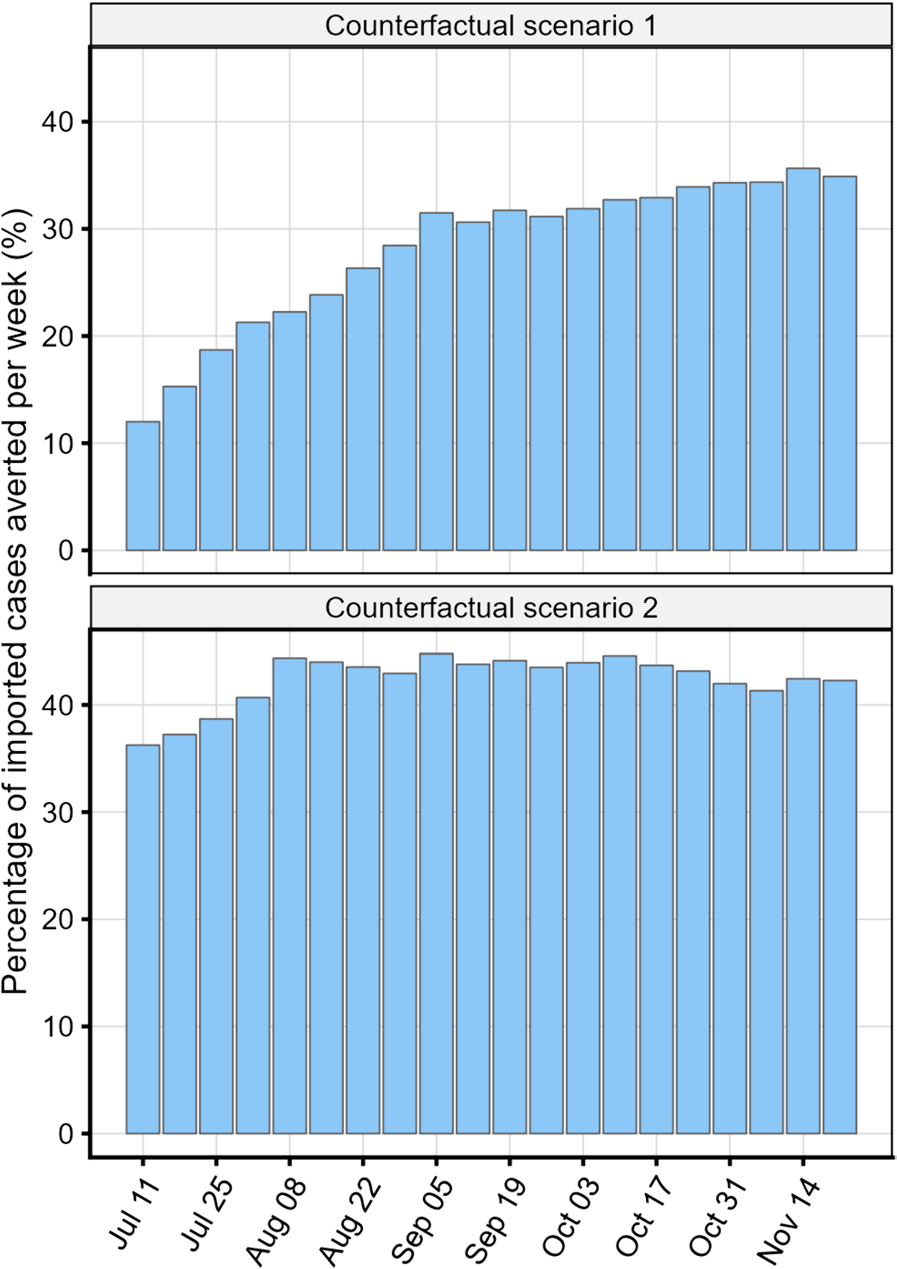
Weekly percentage of infected travellers averted from arriving at Canadian airports from July to November 2021 when comparing the baseline scenario (pre-departure testing of all non-essential travellers) to counterfactual scenario 1 (no pre-departure testing of fully vaccinated non-essential travellers) and to counterfactual scenario 2 (no pre-departure testing of any non-essential travellers)

The counterfactual analysis suggested that pre-departure testing in non-essential travellers reduced importation risk. Compared to the baseline scenario, the risk of importation in non-essential travellers was greater in the counterfactual scenarios, with up to 775 weekly importations (PP ≤ 0.38%) when fully vaccinated travellers were exempt from pre-departure testing (counterfactual scenario 1), and up to 961 weekly imported cases (PP ≤ 0.47%) when all non-essential travellers were exempt from testing (counterfactual 2; Fig. 4). Pre-departure testing in the baseline scenario averted 30% of cases occurring over the study period compared to counterfactual scenario 1, with 12 to 36% of cases prevented weekly (Fig. 5). Even more cases (43%) were prevented when comparing the baseline scenario to counterfactual scenario 2, with 36 to 45% of cases prevented weekly (Fig. 5). The percentage of cases averted in counterfactual scenario 1 increased with time, especially between July and September. For counterfactual scenario 2 the temporal trends on the impact of testing were less pronounced (Fig. 5).

## Discussion

A mathematical model estimating the importation risk of COVID-19 into Canada by combining detailed travel volume data with the evolving global epidemiological landscape and country-specific levels of vaccine- and infection-acquired immunity is presented in this study. The study results suggest that the risk, as measured through the number of travellers arriving infected with SARS-CoV-2 and PP, varied over time by country and Canadian destination airports. Considering the entire study period, the highest overall number of imported COVID-19 cases were estimated to originate from the USA, Mexico, UK, and France. Findings from this study highlight the differential impact of essential and non-essential travellers on COVID-19 importations between July and November 2021. Notably, results from the counterfactual modelling analyses support the effectiveness of pre-departure molecular testing in all non-essential travellers to reduce the number of imported COVID-19 cases.

Flexibility in the model structure and detailed importation risk profiles allow for more nuanced assessments supporting evidence-based policy decision making. By including COVID-19 variant data and detailed travel volumes at the airport level, the model provides a comprehensive characterisation of importation risk by country of departure, variant and point of entry throughout Canada. Furthermore, estimates of importation risk at the airport level allows an evidence-based assessment of the risk and the potential impact on transmission dynamics in the region where the airport is located. In the case of an emergent VOC, the model outputs could be valuable to help target surveillance and on-arrival response efforts towards locations where passengers at higher risk are landing.

Our modelling approach enabled a comprehensive understanding of importation risk through two measures. The PP represents the mean individual-level probability of importation for a given traveller group or country. The number of imported cases provides insight on the level of risk that the traveller group or country poses to Canada by considering the relative importance of both PP and travel volume. The distinction in measures helps interpret the potential roles of different traveller groups or countries on importation risk. For example, model results suggest that essential travellers had a substantially higher PP than non-essential travellers during the study period. This difference can largely be attributed to pre-departure testing requirements for non-essential travellers as supported by results from the counterfactual analyses. However, despite higher PP in essential travellers than non-essential travellers, the overall number of imported cases from essential travellers was low because there were far fewer essential travellers. Another example from the country-level perspective has the opposite conclusion. Model output indicated that travellers from the USA contributed the highest number of imported cases because travel volumes from the USA were higher than any other country, despite the PP of travellers from the USA being lower compared to other countries (e.g. Mexico, Brazil). We found using both measures together is more revealing of importation risk than relying on one alone.

As in [41], which demonstrates the effectiveness of the pre-departure testing program, our model suggests that there would have been nearly twice as many importations estimated to occur in the absence of a pre-departure testing requirement (counterfactual scenario 2). It is important to note that model results are expected to be conservative in terms of the impact of pre-departure testing, given that the mean test sensitivity chosen in our model (i.e. 60%) fell on the lower range of plausible values. The temporal increase in the surplus cases that would have occurred had non-essential fully vaccinated travellers not undergone pre-departure testing (counterfactual scenario 1) can be attributed in part to a growing proportion of non-essential travellers becoming fully vaccinated through time. With vaccination, a larger number of travellers were exempt from the pre-departure testing requirement in counterfactual scenario 1, resulting in increased importations compared to the baseline scenario. The observed temporal increase in fully vaccinated travellers could be explained by the following factors: 1) increased second dose uptake within the Canadian population [42], 2) permitting fully vaccinated non-essential citizens and permanent residents of the US with a GoC approved vaccine to enter Canada for discretionary travel, with exceptions, effective August 9, 2021, and 3) extending factor #2 on September 7, 2021 to all other countries [8, 14]. Consequently, toward the end of the study period, the difference in the impact of removing pre-departure testing in fully vaccinated non-essential travellers as opposed to all non-essential travellers was relatively small. While this analysis highlights the impact of the pre-departure testing program, it also demonstrates the versatility of the model in assessing and comparing the relative influence of different prevention strategies.

Although evaluating the impact of international COVID-19 importations on the local spread in Canada is beyond the scope of this paper, it has been explored previously in different contexts. Results from modelling studies suggest that case importation may have played an important role in local dynamics during the early phase of the COVID-19 pandemic and for emergent variants [19, 20, 43] or in countries with low prevalence and limited public health measures in place to restrict domestic spread [44]. However, international travel restrictions appear to be less effective once the disease is widespread and outbreaks are self-sustaining in the destination country [43, 45, 46]. In that specific context, imported cases would have a relatively small contribution to local transmission dynamics. As such, the impact of international travel restrictions relies on complex and dynamic factors, and requires evaluation and adaptation to the evolving local and global epidemiological situation, while also taking into account their economic and social costs. Previous work evaluating the potential impact of the border re-opening on disease spread within Canada [47] has been performed using an agent-based model [48–51]. However, further analyses would be needed to fully assess the impact of the pre-departure testing requirements on local transmission dynamics among the Canadian population.

Despite the strengths of our modelling approach there are important limitations to consider. First, for the study presented, we did not have access to border testing data for validating model results. Furthermore, as with any highly data driven model, error in input data will decrease accuracy of model output. For instance, the combination of multiple datasets to obtain air travel volume could have led to biased model inputs by traveller group. However, these data sources had the advantage of accounting for Notices to Airmen (NOTAMs) on flight suspensions from specific countries during the study period. Furthermore, the model relies on robust global surveillance data. Poor data quality and quantity can result in biased outcomes, especially in countries with limited testing capacities and unreliable reporting systems. A strength of the current model is the incorporation of a modified semi-Bayesian probabilistic bias approach, implemented to correct the number of reported cases by adjusting for under-ascertainment [27]. Although the country-specific case count estimates from this methodology align well with other published estimates (Fig. A1 in Additional file 1), a minimal amount of data is still required to produce reliable results.

Other limitations arise from the model assumptions. First, by assuming that there was complete protection against reinfection and no-waning in post-infection- and vaccine-induced immunity, model output could underestimate importation risk. Secondly, it was assumed that Canadian travellers only visit one country (the country of departure) and for a limited period prior to departure for Canada and that foreign travellers remain in their respective country of departure without travelling to other countries throughout the pandemic. We justify these assumptions because travel was greatly reduced during the pandemic [52, 53]. Also, we erred on a simplified model structure in the absence of having complete data on travel history prior to departure for Canada. These assumptions likely reduced the accuracy in estimating travellers’ probabilities for vaccine- (for foreign essential travellers) and infection-acquired protection (for all travellers) and probabilities of exposure in the country of departure prior to travel. It is however difficult to know if the resulting error over- or underestimated importation risk. Finally, the model assumed that the traveller population was represented by the underlying country population in terms of the vaccination coverage (for essential travellers only), age demographics and socio-economic landscape, which could potentially lead to bias in terms of estimated exposure risk. For instance, travellers departing from countries with large wealth and income inequalities may have higher quality housing (i.e. less overcrowding) and better access to vaccination, and hence lower SARS-CoV-2 exposure compared to the general population from which model estimates for infection probabilities were calculated [54].

## Conclusions

Our mathematical model provided a detailed COVID-19 importation risk profile for air travellers arriving at Canadian airports from international departures. Model outputs indicated travel groups and countries contributing high importation risk as measured by the number of imported cases and PP. Essential travellers were estimated to contribute fewer importations than non-essential travellers. Furthermore, model results suggest that pre-departure molecular testing in non-essential travellers likely led to lower numbers of imported cases and PP than when compared to counterfactual scenarios that were more lenient. The model we present here was applied to a Canadian COVID-19 context, including an assessment of pre-departure testing, but could be adapted to other similar infectious diseases and border measures, such as vaccination mandates on specific traveller groups and flight suspensions from high-risk countries. As the rate of emerging infectious diseases continues to increase with global environmental change [55], versatile tools such as this importation risk model can help support evidence-based border policy development.

### Supplementary Information

**Additional file 1. **This file contains a detailed description of the methods, data sources and additional results.

## Data Availability

The data that support the findings of this study are available from CBSA, IATA, and GISAID but restrictions apply to the availability of these data, which were used under license for the current study, and so are not publicly available. Data are however available from the authors upon reasonable request and with permission of CBSA, IATA, and GISAID.

## Abbreviations

CBSA: Canada Border Services Agency
IATA: International Air Transport Authority
GoC: Government of Canada
GNI: Gross National Income
OWD: Our World in Data
PP: Percent Positivity
VOC: Variants of concern
VOI: Variants of interest
NOTAM: Notice to Airmen
