# Supplementary material for: A methodology for estimating SARS-CoV-2 importation risk by air travel into Canada between July and November 2021
Source: BMC Public Health. 2024 Apr 19;24:1088. doi: 10.1186/s12889-024-18563-1 (PMC11027292; doi:10.1186/s12889-024-18563-1)
Supplement: Supplementary file 1 — Additional file 1. This file contains a detailed description of the methods, data sources and additional results. [file 12889_2024_18563_MOESM1_ESM.docx]

***Supplementary material***

Table of Contents

[Table of figures 3](#_Toc145004395)

[Table of tables 3](#_Toc145004396)

[1 Border measures implemented in Canada 4](#_Toc145004397)

[Modelling details 4](#_Toc145004398)

[Adjustment to CBSA air travel volumes 4](#_Toc145004399)

[Details for traveller groups 6](#_Toc145004400)

[Underreporting correction factor 6](#_Toc145004401)

[Post-infection and vaccination immunity 9](#_Toc145004402)

[Daily probability of infection 11](#_Toc145004403)

[2 Derivation of probability of importation equations 11](#_Toc145004404)

[Probability of importation: essential travellers 11](#_Toc145004405)

[2.1.1 Canadian traveller infection probabilities 11](#_Toc145004406)

[2.1.2 Foreign traveller infection probabilities 11](#_Toc145004407)

[Probability of importation: Non-essential travellers 12](#_Toc145004408)

[2.1.3 Canadian traveller infection probabilities 12](#_Toc145004409)

[2.1.4 Foreign traveller infection probabilities 14](#_Toc145004410)

[Calculating the number of infected passengers by variant 15](#_Toc145004411)

[3 References 17](#_Toc145004412)

# **Table of figures**

[**Figure A1** Estimates of the “true” case counts using a Bayesian method developed for this study compared to estimates from two published “S-E-I-R” models (from Imperial College London and Institute for Health Metrics and Evaluation) for select countries between March 2020 and December 2021. 7](#_Toc158965714)

[**Figure A2:** Distribution of imported variants among the top ten countries most at risk of COVID-19 importation per week. 15](#_Toc158965715)

# **Table of tables**

[**Table A1.** Weekly scaling factor describing the percent reduction in Canadian Border Services Agency (CBSA) travel volumes between July 11-November 27, 2021 (9). 4](#_Toc158965716)

[**Table A2.** Mean vaccine effectiveness, VE, against SARS-CoV-2 infection for non-essential and essential travellers given their country of residence and vaccine status (GoC-approval status and partially vs fully vaccinated). 9](#_Toc158965717)

1. Border measures implemented in Canada

The first reported case of COVID-19 in Canada was on January 27, 2020 in a traveller returning from Wuhan (1). During 2020 and 2021, the Government of Canada (GoC) implemented multiple types of international border measures as summarised in Figure 1 of the main manuscript (2-8).

## Modelling details

The model estimates the expected number of air passengers infected with SARS-CoV-2 arriving in Canada by country of departure, destination airport and variant based on: 1) air travel volumes, 2) traveller groups: a) reason for travel (essential or non-essential), b) nationality (Canadian citizen and residents or foreign nationals), and c) vaccine status (unvaccinated, partially or fully vaccinated with GoC, or non-GoC vaccines), 3) natural immunity and vaccine coverage, 4) case under-reporting from country of travel origin, and 5) country-specific daily probabilities of infection.

## Adjustment to CBSA air travel volumes

Daily air travel volumes expected into Canada were provided by the Canadian Border Services Agency (CBSA) from the Advanced Passenger Information (API) database (9). While these data describe the expected travel volumes to all airports in Canada from the country of departure (i.e. the country from which travel to Canada was initiated), they are not the actual count of arriving passengers, and may overestimate the actual travel volume due to cancelled, delayed, or missed flights. To correct for this overestimation, the weekly percent reduction (ranging between 14-30% (Table A1)) between the overall API travel volume and the actual number of travellers who entered Canada by air (derived from passage data provided by the CBSA) was applied uniformly to the API travel volume for each country of departure.

**Table A1.** Weekly scaling factor describing the percent reduction in Canadian Border Services Agency (CBSA) travel volumes between July 11-November 27, 2021 (9).

| **Date** | **Scaling factor (%)** | **Date** | **Scaling factor (%)** |
| --- | --- | --- | --- |
| July 11 | 28 | September 19 | 18 |
| July 18 | 29 | September 26 | 18 |
| July 25 | 28 | October 3 | 17 |
| August 1 | 27 | October 10 | 16 |
| August 8 | 22 | October 17 | 16 |
| August 15 | 22 | October 24 | 17 |
| August 22 | 20 | October 31 | 17 |
| August 29 | 19 | November 7 | 16 |
| September 5 | 19 | November 14 | 15 |
| September 12 | 18 | November 21 | 14 |

## Details for traveller groups

**Essential and non-essential travellers**

The number of entries for non-essential travellers into Canada reported in the ArriveCan and ContactTrace data collection platforms (10, 11), as well as CBSA travel volume, was used to calculate the weekly and country-specific proportion of *non-essential* travellers (Eq. 1), which ranged from 77-89% between July 11 and November 27, 2021.

$$proportion non-essential = \frac{{Number non-essential}_{ArriveCan + Contact Trace}}{Total travel volume_{CBSA}} (1)$$

Using similar methodology to Johansson et al. (12) and test sensitivity parameters of the molecular pre-departure test informed by (13) (i.e. 90% of detecting an infection with 10^4^ copies/ml), the modelled test sensitivity for non-essential travellers, *se*, was set at 60% to account for variation in the sensitivity with respect to time since infection.

**Canadian and foreign residents**

The weekly proportions of Canadian and foreign residents were calculated differently for essential and non-essential travellers. For *essential* travellers, the issuing country of the travel document presented at the time of ticket purchase from the CBSA’s API database was used as a proxy to calculate the country-level proportion of Canadian and foreign residents. For *non-essential* travellers, travellers in the ArriveCan and ContactTrace databases who indicated that they were “returning to Canada” were defined as Canadian residents*,* while all other travellers were proxied by the term foreign resident*.*

**Vaccination status**

Travel groupings by vaccination status (unvaccinated, partially or fully vaccinated) for either GoC or non-GoC approved vaccines, were used to define vaccine effectiveness parameters and the probability of vaccine-acquired immunity in the model (described in the “Traveller groups” and “Model formulation” sections of the manuscript, and “Post-infection and vaccination immunity” of this document).

## Underreporting correction factor

We adapted the method by Wu et al. (2020) (14) to correct for underreporting in cases. In this method a semi-Bayesian probabilistic bias analysis estimates the actual number of cases in a region based on the population size, reported cases, and reported number of COVID-19 tests. We adapted the method to account for the temporal decrease in the susceptible population due to increasing cumulative case counts and the time-varying vaccine coverage. Finally, a correction factor,$CF_{c,t}$,$CF_{t}$ for a given country $c$ during a specific time period $t$ could be calculated by dividing the number of estimated cases by the number of reported cases.

Daily country- and state-specific data on the number vaccinated (15-17), the number tested (15, 16, 18, 19), and the number of new cases (15, 16, 19, 20) were used to calculate the$CF_{c,t}$ from March 2020 onwards$CF_{t} from March 2020 onwards.$. Linear interpolation was used to estimate the number tested or vaccinated in the case of missing data. As described in (14), the estimation method provides unstable results for settings with low testing rates. To circumvent this issue and since substantial data was unavailable early in the pandemic, the data were aggregated from March-August 2020, and in one-month intervals thereafter.

The susceptible population size for each time period for a given country *c­* was calculated as a function of the 2020 population estimates ($Pop_{c,2020}$) (15, 21, 22), the reported proportion protected against infection and the proportion of people successfully vaccinated (${PropVacc}_{c,d}$ $\mathrm{Vacc}_{c,d},$*,* see section “Post-infection and vaccination immunity”, Eq.3, and Table A2):

${SPop}_{c,d}=Pop_{c,2020} \times\left( 1-\frac{{InfectionsReported}_{c ,d-1}}{Pop_{c ,2020}} \right) \times\left( 1- {PropVacc}_{c,d} \right)$ (2)

In rare instances, the estimated $CF_{c,t}$ value was below 1, in which case a ratio of 1 was assumed. Based on a visual exploration of the estimated $CF_{c,t}$values, a maximum threshold value of 80 was chosen and any estimated value above this threshold was discarded. Any missing $CF_{c,t}$ values due to data filtering with respect to the threshold or insufficient data were replaced with the median value across all available $CF_{c,t}$ for the respective country or state, whenever possible.

For countries that did not have case, testing or vaccination data, a regression modelling approach was used to impute their $CF_{c,t}$ value. The optimal model structure was determined by regressing the available $CF_{c,t}$ estimates on the following country-specific predictors: a) Detection and Reporting score (DRS) from the 2019 Global Health Security index ((23); as a measure of the capacity of the country to detect and report infectious disease cases), and b) the 2019 Growth National Income (GNI) (24) per capita (as a proxy for the effectiveness of the surveillance system to detect, test and report COVID-19 cases). The functional form of the variables was derived with the response variable and it was verified that they did not violate model assumptions of linearity. The regression model was run for each time period between March 2020 and February 2021, and the average Akaike’s information criterion (AIC) for the entire period was computed. The best model was selected as having the lowest mean AIC and residuals that conformed to the parametric distribution. The predictor in the final model was a log-transformed value of GNI per capita.

The quality in the estimated number of true cases was visually assessed against other modelled estimates on case counts from Medical Research Council Centre for Global Infectious Disease Analysis and the Institute for Health Metrics and Evaluation {Institute for Health Metrics and Evaluation (IHME), 2022 #136}{Institute for Health Metrics and Evaluation (IHME), 2022 #136}(15, 25, 26){Institute for Health Metrics and Evaluation (IHME), 2022 #136}. For the visual assessment we selected 12 countries that ranged in their concordance with the other modelled estimates. We find there is generally good agreement between our estimated number of true cases, given the $CF_{c,t}$, and other published counts (Figure A1).

| 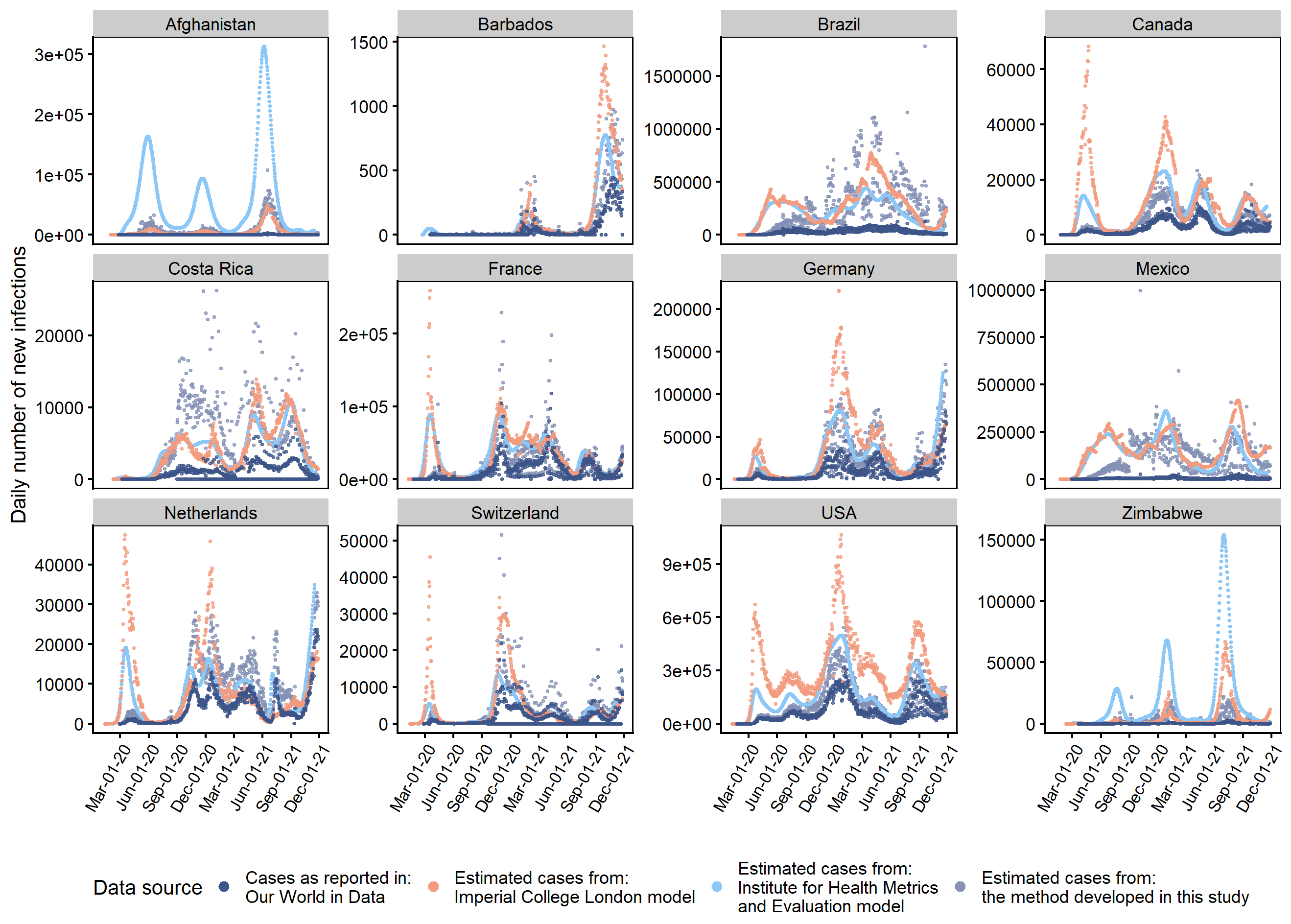 |
| --- |

**Figure A1** Estimates of the “true” case counts using a Bayesian method developed for this study compared to estimates from two published “S-E-I-R” models (from Imperial College London and Institute for Health Metrics and Evaluation) for select countries between March 2020 and December 2021.

## Post-infection and vaccination immunity

The probability of a traveller importing infection into Canada from country of departure $cd$ depends in part on whether they have acquired protection from a previous infection or vaccination since the start of the pandemic. Assuming that previous infections provide complete immunity against re-infection, the proportion of people who are protected against infection on day *d* in a given country $c$ due to a previous infection is equal to:

$${PropInf}_{c ,d}=\frac{{InfectionsReported}_{c ,d-1}\times CF_{c,t}}{Pop_{c ,2020}} (3)$$

Where ${InfectionsReported}_{c ,d-1}$is the number of reported infections prior to day *d*, ${CF}_{c,t}$ is the time-varying country-specific case correction factor (see section “Underreporting correction factor”), and $Pop_{c ,2020}$ is the 2020 population size in country $c$. The probability that a traveller from country of residence $cr$ has acquired protection against infection in $cr$ on day $d$ due to previous infection, ${Pinf}_{cr ,d}$, is equal to ${PropInf}_{cr ,d}$.

Conversely, vaccination was assumed to provide partial immunity against infection; vaccine effectiveness corresponds to the probability that a vaccinated individual develops complete immunity against infection (i.e. 0% probability of getting infected). The term “successfully vaccinated” is hereafter used to describe a traveller that has been vaccinated and has successfully developed immunity against infection given the vaccine effectiveness for the assumed vaccination status.

To account for variability in the effectiveness between the vaccine candidates that were used across the world, it was assumed that the vaccine effectiveness against infection follows a normal distribution with a standard deviation of 1.5% (mean values are provided in Table A2). A slightly higher vaccine effectiveness was used for Canada and the United States of America (USA) since the majority of vaccines administered in these countries were of the type molecular ribonucleic acid (mRNA), which has been found to provide better protection than other vaccines (27).

The proportion of people successfully vaccinated on day *d* in country $c$ was assumed to be proportional to the vaccine coverage ($Prop_{c,d})$, and vaccine effectiveness (${VE}_{c}$) by vaccine status (Table A2):

${PropVacc}_{c,d}=Prop_{c,d, partially}\times{VE}_{c,partially}+ Prop_{c,d, fully}\times{VE}_{c,fully} \left( 4 \right)$

The vaccine coverage in each country was extracted from an openly available dataset (15). Given that data regarding vaccine status is available for *non-essential* travellers*,* the probability of these travellers being successfully vaccinated, ${Pvacc\_NE}_{cr,status}$, is equal to the vaccine effectiveness for the corresponding vaccine status in country $cr$ (Table A2). In contrast, the probability that an *essential* traveller from country of residence $cr$ successfully acquired protection against infection on or prior to day $d$ due to vaccination, ${Pvacc\_E}_{cr,d}$, is equal to ${PropVacc}_{cr,d}$.

**Table A2.** Mean vaccine effectiveness, VE, against SARS-CoV-2 infection for non-essential and essential travellers given their country of residence and vaccine status (GoC-approval status and partially vs fully vaccinated).

| **Vaccine status: GoC-approval** | **Country of residence** | **Assumption** | **Vaccine status: doses received** | **Mean VE estimate** | **Reference** |
| --- | --- | --- | --- | --- | --- |
| ***Non-essential* travellers^ⱡ^** | | | | | |
| Non GoC-approved | a) All countries | Assumed to be equal to VE for AZ | Partially vaccinated^Δ^ | 0.30 | (27-29) |
|  |  |  | Fully vaccinated | 0.60 | (27-30) |
| GoC-approved | b) USA and Canada | Assumed to be equal to VE for mRNA vaccines | Partially vaccinated^Δ^ | 0.35 | (29) |
|  |  |  | Fully vaccinated | 0.82 | (29, 31) |
|  | c) Non-USA foreign countries | Assumed to be equal to the average of AZ VE (a) and mRNA VE (b) | Partially vaccinated^Δ^ | 0.32 | calculated |
|  |  |  | Fully vaccinated | 0.71 |  |
| Mixture of GoC- and non GoC-approved | d) All countries | Assumed to be equal to the average of non GoC-approved VE (a) and GoC-approved VE (c) | Fully vaccinated | 0.66 | calculated |
| ***Essential* travellers*** | | | | | |
| Data unavailable | e) USA and Canada | Assumed to be equal to VE for mRNA vaccines (a) | Partially vaccinated | 0.35 | (29) |
|  |  |  | Fully vaccinated | 0.82 | (29, 31) |
| Data unavailable | f) Non-USA foreign countries | Assumed to be equal to the average of non GoC-approved VE (a) and GoC-approved VE (c) | Partially vaccinated | 0.31 | calculated |
|  |  |  | Fully vaccinated | 0.66 |  |

AZ = Astrazeneca; GoC = Government of Canada; VE = Vaccine effectiveness

**^ⱡ^** The proportion of travellers in each vaccine status category were based on ArriveCan and ContactTrace

^*^ The proportion of travellers in each vaccine status category were based on country-specific vaccine coverage data (15)

^Δ^ Includes fully vaccinated travellers that received their second dose within 14 days of travel to Canada

## Daily probability of infection

The daily probability of infection among susceptible individuals is a product of the number of new reported cases in country $cd$ on day *d*,$\mathrm{NewCases}_{c,d}$ $\mathrm{NewCases}_{\mathrm{cd},d}$*,* and the underreporting correction factor for the corresponding time period, $CF{}_{cd,t}$, over the susceptible population (i.e. the proportion of the population that was not protected against infection due to previous infection ($({1-PropInf}_{cd,d}$); Eq. 3) and/or vaccination ( $({1-PropVacc}_{cd,d}$); Eq. 4) and the 2020 country-specific population size, $\mathrm{Po}p_{c,2020}$ $\mathrm{Po}p_{cd,2020}$):

$$\beta_{cd,d}=\frac{{NewCases}_{cd,d}*CF_{cd,t}}{Pop_{cd, 2020}*\left( 1- {PropInf}_{cd,d} \right)*(1-{PropVacc}_{cd,d})} (5)$$

1. Derivation of probability of importation equations

For the following analyses, it was assumed that day 0 represents the start of the pandemic and that day *s* is the day at which the individual travels from their country of departure to Canada (i.e. *s* is the number of days between the start of the pandemic and the travel date to Canada). All events (e.g. arrival in country $cd$ for *CNDs*, travel to Canada, and pre-departure testing) were assumed to take place at the start of the given day. The sum of the latent and infectious periods is represented by the parameter *n* in the equations below.

## Probability of importation: essential travellers

- - 1. Canadian traveller infection probabilities

It is assumed that a CND that has developed immunity following vaccination or infection prior to the date of departure from Canada to country $cd$ cannot import infection from country $cd$. When the time spent in country $cd$ is less than the sum of the latent and infectious periods (i.e. $\boldsymbol{t}_{\boldsymbol{c}}\boldsymbol{\leq n}$), the probability of a CND importing infection from country $cd$ into Canada on day *s* is equal to the probability of that person getting infected on any day during the trip, multiplied by the probability of not having been infected prior to the trip and not having been successfully vaccinated in their country of residence, Canada ($CAN$):

$${\boldsymbol{P}\boldsymbol{\_}\boldsymbol{E}}_{\boldsymbol{s}\boldsymbol{,}\boldsymbol{cd}}\boldsymbol{=}\left( \boldsymbol{1}\boldsymbol{-}\prod_{\boldsymbol{d}\boldsymbol{=}{\boldsymbol{s}\boldsymbol{-} \boldsymbol{t}}_{\boldsymbol{c}}}^{\boldsymbol{s}\boldsymbol{-}\boldsymbol{1}} \left( \boldsymbol{1}\boldsymbol{-}\boldsymbol{\beta}_{\boldsymbol{cd}\boldsymbol{,}\boldsymbol{d}} \right) \right)\boldsymbol{\times}\left( \boldsymbol{1}\boldsymbol{-}\boldsymbol{Pinf}_{\boldsymbol{CAN}\boldsymbol{,}\boldsymbol{s}\boldsymbol{-}\left( \boldsymbol{t}_{\boldsymbol{c}}\boldsymbol{+}\boldsymbol{1} \right)} \right)\boldsymbol{\times}\left( \boldsymbol{1}\boldsymbol{-} \boldsymbol{Pvacc}_{\boldsymbol{CAN}\boldsymbol{,}\boldsymbol{s}\boldsymbol{-}\left( \boldsymbol{t}_{\boldsymbol{c}}\boldsymbol{+}\boldsymbol{1} \right)} \right)\boldsymbol{(}\boldsymbol{6}\boldsymbol{)}$$

When $\boldsymbol{t}_{\boldsymbol{c}}\boldsymbol{>n}$ , the probability of a CND importing infection from country $cd$ into Canada on day *s* is equal to the sum of the probability of not getting infected during $t_{c}$ and the probability of getting infected in the country, recovering and becoming immune before departure multiplied by the probability of not having been infected in Canada prior to the trip and not having been successfully vaccinated in Canada:

$${\boldsymbol{P}\boldsymbol{\_}\boldsymbol{E}}_{\boldsymbol{s}\boldsymbol{,}\boldsymbol{cd}}\boldsymbol{=}\left( \prod_{\boldsymbol{d}\boldsymbol{=}{\boldsymbol{s}\boldsymbol{-} \boldsymbol{t}}_{\boldsymbol{c}}}^{\boldsymbol{s}\boldsymbol{-}\left( \boldsymbol{n}\boldsymbol{+}\boldsymbol{1} \right)} \left( \boldsymbol{1}\boldsymbol{-}\boldsymbol{\beta}_{\boldsymbol{cd}\boldsymbol{,}\boldsymbol{d}} \right)\boldsymbol{-}\prod_{\boldsymbol{d}\boldsymbol{=}{\boldsymbol{s}\boldsymbol{-} \boldsymbol{t}}_{\boldsymbol{c}}}^{\boldsymbol{s}\boldsymbol{-}\boldsymbol{1}} \left( \boldsymbol{1}\boldsymbol{-}\boldsymbol{\beta}_{\boldsymbol{cd}\boldsymbol{,}\boldsymbol{d}} \right) \right)\boldsymbol{\times}\left( \boldsymbol{1}\boldsymbol{-}\boldsymbol{Pinf}_{\boldsymbol{CAN}\boldsymbol{,}\boldsymbol{s}\boldsymbol{-}\left( \boldsymbol{t}_{\boldsymbol{c}}\boldsymbol{+}\boldsymbol{1} \right)} \right)\boldsymbol{\times}\left( \boldsymbol{1}\boldsymbol{-} \boldsymbol{Pvacc}_{\boldsymbol{CAN}\boldsymbol{,}\boldsymbol{s}\boldsymbol{-}\left( \boldsymbol{t}_{\boldsymbol{c}}\boldsymbol{+}\boldsymbol{1} \right)} \right)\boldsymbol{(}\boldsymbol{7}\boldsymbol{)}$$

- - 1. Foreign traveller infection probabilities

It is assumed that the time that a FT has spent in country $cd$ (i.e. time from the start of the pandemic to date) will always be larger than the sum of the latent and infectious periods (i.e*.* $t_{c}>n$ $t_{c}>n$). The probability of a traveller entering Canada infected is therefore equal to the probability of getting infected on any of the *n* days prior to travel to Canada, multiplied by the probability of not having been infected and not having been successfully vaccinated in country $cd$ prior to this *n*-day period. The probability of a FT travelling from country $cd$ to Canada on day *s* infected is therefore equal to:

$${\boldsymbol{P}\boldsymbol{\_}\boldsymbol{E}}_{\boldsymbol{s}\boldsymbol{,}\boldsymbol{cd}}\boldsymbol{=}\left( \boldsymbol{1}\boldsymbol{-}\prod_{\boldsymbol{d}\boldsymbol{=}\boldsymbol{s}\boldsymbol{-}\boldsymbol{n}}^{\boldsymbol{s}\boldsymbol{-}\boldsymbol{1}} \left( \boldsymbol{1}\boldsymbol{-}\boldsymbol{\beta}_{\boldsymbol{cd}\boldsymbol{,}\boldsymbol{d}} \right) \right)\boldsymbol{\times}\left( \boldsymbol{1}\boldsymbol{-}\boldsymbol{Pinf}_{\boldsymbol{cd}\boldsymbol{,}\boldsymbol{s}\boldsymbol{-}\left( \boldsymbol{n}\boldsymbol{+}\boldsymbol{1} \right)} \right)\boldsymbol{\times}\left( \boldsymbol{1}\boldsymbol{-} \boldsymbol{Pvacc}_{\boldsymbol{cd}\boldsymbol{,}\boldsymbol{s}\boldsymbol{-}\left( \boldsymbol{n}\boldsymbol{+}\boldsymbol{1} \right)} \right)\boldsymbol{(}\boldsymbol{8}\boldsymbol{)}$$

## Probability of importation: Non-essential travellers

For a *non-essential* traveller, infection is imported into Canada when a person travelling to Canada is infected on test day but has a false negative result or is not infected or immune on test day but gets infected during the remaining days prior to departure. The probability of a *non-essential* (NE) individual travelling by air from country $cd$ and arriving infected in Canada on day *s* is therefore equal to:

$$P\_NE=P\_test\_Inf \times\left( 1-se \right) + P\_test\_NInf \times P\_aftertest\_Inf (9)$$

Where $P_{testday\_infect}$ $P\_test\_Inf$ is the probability of being infected on test day, $1-se$ is the probability of getting a negative test result given infection on test day, $P_{testday\_no\_infect}$ $P\_test\_NInf$ is the probability of not being infected on test day for a person that is not immune through vaccination or prior infection and $P\_aftertest\_Inf$ is the probability of becoming infected after the test day.

- - 1. Canadian traveller infection probabilities

It is assumed that a CND that has developed immunity following vaccination or infection prior to the date of departure from Canada to country $cd$ cannot import infection from country $cd$.

When the time in country $cd$ is less than or equal to the sum of the latent and infectious periods (i.e. $t_{c}\leq n+\mu$ $t_{c}\leq n$ ), the probability of a CND being infected on test day is equal to the probability of getting infected on any given day during *t_c_* before the test day multiplied by the probability of not having been infected and not having been successfully vaccinated in Canada prior to the trip (similar to Eq. 6):

$${P\_test\_Inf}_{\boldsymbol{s}\boldsymbol{,}\boldsymbol{cd}\boldsymbol{,}\boldsymbol{status}}= \left( 1-\prod_{d={s- t}_{c}}^{s-(\mu+1)} \left( 1-\beta_{cd,d} \right) \right)\boldsymbol{\times}\left( 1-{Pinf}_{CAN,s-\left( t_{c}+1 \right)} \right)\times\left( 1- {Pvacc}_{CAN,status} \right) (10)$$

The probability of not being infected on test day because the individual did not acquire infection on any day during the trip prior to the test and the person did not develop immunity through infection or vaccination in Canada prior to the trip to country $cd$ is:

$${P\_test\_NInf}_{\boldsymbol{s}\boldsymbol{,}\boldsymbol{cd}\boldsymbol{,}\boldsymbol{status}}=\prod_{d={s- t}_{c}}^{s-(\mu+1)} \left( 1-\beta_{cd,d} \right) \times\left( 1-{Pinf}_{CAN,s-\left( t_{c}+1 \right)} \right)\times\left( 1- {Pvacc}_{CAN,status} \right) (11)$$

The probability of acquiring infection on any of the remaining days of the trip prior to departure to Canada is:

$${P\_aftertest\_Inf}_{\boldsymbol{s}\boldsymbol{,}\boldsymbol{cd}}= 1-\prod_{d=s-\mu}^{s-1} \left( 1-\beta_{cd,d} \right) (12)$$

Therefore, based on Eq. 9, when $t_{c}\leq n+\mu$ $t_{c}\leq n$ , the probability of a CND importing infection from country $cd$ into Canada on day *s* is:

$${P\_NE}_{s,cd,status}= \left[ \left( 1-\prod_{d={s- t}_{c}}^{s-(\mu+1)} \left( 1-\beta_{cd,d} \right) \right)\left( 1-se \right) + \left( \prod_{d={s- t}_{c}}^{s-(\mu+1)} \left( 1-\beta_{cd,d} \right) \right)\left( 1-\prod_{d=s-\mu}^{s-1} \left( 1-\beta_{cd,d} \right) \right) \right] \times\left[ \left( 1-{Pinf}_{CAN,s-\left( t_{c}+1 \right)} \right)\times\left( 1- {Pvacc}_{CAN,status} \right) \right] (13)$$

Which simplifies to:

$${\boldsymbol{P}\boldsymbol{\_}\boldsymbol{NE}}_{\boldsymbol{s}\boldsymbol{,}\boldsymbol{cd}\boldsymbol{,}\boldsymbol{status}}\boldsymbol{=}\left[ \boldsymbol{1}\boldsymbol{-}\boldsymbol{se}\boldsymbol{+}\boldsymbol{se}\prod_{\boldsymbol{d}\boldsymbol{=}{\boldsymbol{s}\boldsymbol{-} \boldsymbol{t}}_{\boldsymbol{c}}}^{\boldsymbol{s}\boldsymbol{-}\boldsymbol{(\mu+}\boldsymbol{1}\boldsymbol{)}} \left( \boldsymbol{1}\boldsymbol{-}\boldsymbol{\beta}_{\boldsymbol{cd}\boldsymbol{,}\boldsymbol{d}} \right)\boldsymbol{-}\prod_{\boldsymbol{d}\boldsymbol{=}{\boldsymbol{s}\boldsymbol{-} \boldsymbol{t}}_{\boldsymbol{c}}}^{\boldsymbol{s}\boldsymbol{-}\boldsymbol{1}} \left( \boldsymbol{1}\boldsymbol{-}\boldsymbol{\beta}_{\boldsymbol{cd}\boldsymbol{,}\boldsymbol{d}} \right) \right]\boldsymbol{\times}\left( \boldsymbol{1}\boldsymbol{-}\boldsymbol{Pinf}_{\boldsymbol{CAN}\boldsymbol{,}\boldsymbol{s}\boldsymbol{-}\left( \boldsymbol{t}_{\boldsymbol{c}}\boldsymbol{+}\boldsymbol{1} \right)} \right)\boldsymbol{\times}\left( \boldsymbol{1}\boldsymbol{-}\boldsymbol{Pvacc}_{\boldsymbol{CAN}\boldsymbol{,}\boldsymbol{status}} \right)\boldsymbol{(}\boldsymbol{14}\boldsymbol{)}$$

When time in country $cd$ is larger than the sum of latent and infectious periods (i.e. $t_{c}>n +\mu$ $t_{c}>n$ ), the probability of an individual being infected on test day (and remaining infectious on travel day to Canada) is equal to the probability of getting infected during the $\left( n-\mu\right)$ days prior to the test given that the person did not get infected during the previous days of the trip (i.e. any day from the beginning of the trip to the *n*-day period prior to departure for Canada), multiplied by the probability of not having been infected in Canada prior to the trip and not having been successfully vaccinated in Canada (similar to Eq. 7):

$${P\_test\_Inf}_{\boldsymbol{s}\boldsymbol{,}\boldsymbol{cd}\boldsymbol{,}\boldsymbol{status}}=\left( \prod_{d={s- t}_{c}}^{s-(n+1)} (1-\beta_{cd,d})-\prod_{d={s- t}_{c}}^{s-(\mu+1)} \left( 1-\beta_{cd,d} \right) \right)\times\left( 1-{Pinf}_{CAN,s-\left( t_{c}+1 \right)} \right)\times\left( 1- {Pvacc}_{CAN,status} \right) (15)$$

The probability of not being infected on test day because the individual did not acquire infection on any day during the trip prior to the test, and the person did not develop immunity through vaccination or infection in Canada is:

$${P\_test\_NInf}_{\boldsymbol{s}\boldsymbol{,}\boldsymbol{cd}\boldsymbol{,}\boldsymbol{status}}=\left( \prod_{d={s- t}_{c}}^{s-(\mu+1)} \left( 1-\beta_{cd,d} \right) \right)\times\left( \left( 1-{Pinf}_{CAN,s-\left( t_{c}+1 \right)} \right)\times\left( 1- {Pvacc}_{CAN,status} \right) \right) (16)$$

The probability of acquiring infection on any of the remaining days of the trip prior to departure to Canada, ${P\_aftertest\_Inf}_{\boldsymbol{s,cd}}$, is represented in Eq. 12.

Therefore, based on Eq. 9, when $t_{c}>n+\mu$ $t_{c}>n$ *,* the probability of a CND importing infection from country $cd$ into Canada on day *s* is:

$${P\_NE}_{s,cd,status}= \left[ \left( \prod_{d={s- t}_{c}}^{s-(n+1)} (1-\beta_{cd,d})-\prod_{d={s- t}_{c}}^{s-(\mu+1)} \left( 1-\beta_{cd,d} \right) \right)\left( 1-se \right)+\left( \prod_{d={s- t}_{c}}^{s-(\mu+1)} \left( 1-\beta_{cd,d} \right) \right)\left( 1-\prod_{d=s-\mu}^{s-1} \left( 1-\beta_{cd,d} \right) \right) \right]\times\left( 1-{Pinf}_{CAN,s-\left( t_{c}+1 \right)} \right)\times\left( 1- {Pvacc}_{CAN,status} \right) (17)$$

Which simplifies to:

$$\boldsymbol{P\_NE}_{\boldsymbol{s,cd,status}}\boldsymbol{=}\left[ \left( \boldsymbol{1-se} \right)\prod_{\boldsymbol{d=}\boldsymbol{s- t}_{\boldsymbol{c}}}^{\boldsymbol{s-(n+1)}} \left( \boldsymbol{1-}\boldsymbol{\beta}_{\boldsymbol{cd}\boldsymbol{,d}} \right)\boldsymbol{+se}\prod_{\boldsymbol{d=}\boldsymbol{s- t}_{\boldsymbol{c}}}^{\boldsymbol{s-}\left( \boldsymbol{\mu+1} \right)} \left( \boldsymbol{1-}\boldsymbol{\beta}_{\boldsymbol{cd}\boldsymbol{,d}} \right)\boldsymbol{-}\prod_{\boldsymbol{d=}\boldsymbol{s- t}_{\boldsymbol{c}}}^{\boldsymbol{s-1}} \left( \boldsymbol{1-}\boldsymbol{\beta}_{\boldsymbol{cd}\boldsymbol{,d}} \right) \right]\boldsymbol{\times}\left( \boldsymbol{1-}\boldsymbol{Pinf}_{\boldsymbol{CAN}\boldsymbol{,s-}\left( \boldsymbol{t}_{\boldsymbol{c}}\boldsymbol{+1} \right)} \right)\boldsymbol{\times}\left( \boldsymbol{1-}\boldsymbol{Pvacc}_{\boldsymbol{CAN}\boldsymbol{,status}} \right) (18)$$

- - 1. Foreign traveller infection probabilities

The time that a FT spent in country $cd$, $t_{c}$, is assumed to always be greater than the sum of the latent and infectious periods (i.e. $t_{c}>n+\mu$ $t_{c}>n$ ). The probability of a FT being infected on test day (and remaining infectious on travel day to Canada) is equal to the probability of acquiring infection on any day during the $(n-\mu)$ days prior to the test, multiplied by the probability of not having been infected and the probability of not having been successfully vaccinated in country $cd$ during any of the previous days (i.e. any day from the start of the pandemic to the *n*-day period prior to departure to Canada) (similar to Eq. 8):

$${P\_test\_Inf}_{\boldsymbol{s}\boldsymbol{,}\boldsymbol{cd}\boldsymbol{,}\boldsymbol{status}}= \left( 1-\prod_{d=s-n}^{s-(\mu+1)} \left( 1-\beta_{cd,d} \right) \right)\times\left( \left( 1-{Pinf}_{cd,s-\left( n+1 \right)} \right)\times\left( 1- {Pvacc}_{cd,status} \right) \right) (18)$$

The probability of not being infected on test day because the individual did not acquire infection on any of the $(n-\mu)$ days prior to the test, and the person did not develop immunity through vaccination or infection in country $cd$ is:

$${P\_test\_NInf}_{\boldsymbol{s}\boldsymbol{,}\boldsymbol{cd}\boldsymbol{,}\boldsymbol{status}}=\prod_{d=s-n}^{s-(\mu+1)} \left( 1-\beta_{cd,d} \right) \times\left( \left( 1-{Pinf}_{cd,s-\left( n+1 \right)} \right)\times\left( 1- {Pvacc}_{cd,status} \right) \right) (19)$$

The probability of acquiring infection on any of the remaining days of the trip after the test and prior to departure to Canada, ${P\_aftertest\_Inf}_{\boldsymbol{s,cd}}$, is represented in Eq. 12.

Therefore, based on Eq. 9, the probability of a FT importing infection from country $cd$ into Canada on day *s* is:

$${P\_NE}_{s,cd,status}= \left[ \left( 1-\prod_{d=s-n}^{s-(\mu+1)} \left( 1-\beta_{cd,d} \right) \right)\left( 1-se \right) + \left( \prod_{d=s-n}^{s-(\mu+1)} \left( 1-\beta_{cd,d} \right) \right)\left( 1-\prod_{d=s-\mu}^{s-1} \left( 1-\beta_{cd,d} \right) \right) \right] \times\left( 1-{Pinf}_{cd,s-\left( n+1 \right)} \right)\times\left( 1- {Pvacc}_{cd,status} \right) (20)$$

Which simplifies to:

${\boldsymbol{P}\boldsymbol{\_}\boldsymbol{NE}}_{\boldsymbol{s}\boldsymbol{,}\boldsymbol{cd}\boldsymbol{,}\boldsymbol{status}}\boldsymbol{=}\left[ \boldsymbol{1}\boldsymbol{-}\boldsymbol{se}\boldsymbol{+}\boldsymbol{se}\prod_{\boldsymbol{d}\boldsymbol{=}\boldsymbol{s}\boldsymbol{-}\boldsymbol{n}}^{\boldsymbol{s}\boldsymbol{-}\left( \boldsymbol{\mu+}\boldsymbol{1} \right)} \left( \boldsymbol{1}\boldsymbol{-}\boldsymbol{\beta}_{\boldsymbol{cd}\boldsymbol{,}\boldsymbol{d}} \right)\boldsymbol{-}\prod_{\boldsymbol{d}\boldsymbol{=}\boldsymbol{s}\boldsymbol{-}\boldsymbol{n}}^{\boldsymbol{s}\boldsymbol{-}\boldsymbol{1}} \left( \boldsymbol{1}\boldsymbol{-}\boldsymbol{\beta}_{\boldsymbol{cd}\boldsymbol{,}\boldsymbol{d}} \right) \right]\boldsymbol{\times}\left( \boldsymbol{1}\boldsymbol{-}\boldsymbol{Pinf}_{\boldsymbol{cd}\boldsymbol{,}\boldsymbol{s}\boldsymbol{-}\left( \boldsymbol{n}\boldsymbol{+}\boldsymbol{1} \right)} \right)\boldsymbol{\times}\left( \boldsymbol{1}\boldsymbol{-} \boldsymbol{Pvacc}_{\boldsymbol{cd}\boldsymbol{,}\boldsymbol{status}} \right) \left( 21 \right)$

## Calculating the number of infected passengers by variant

Model output was aggregated by variants of concern (VOC) and variants of interest (VOI) (Figure A2). The VOC and VOI data were downloaded from the GISAID EpiFlu™ Database (32) in accordance with the GISAID Access Agreement of data sharing terms (https://www.gisaid.org/registration/terms-of-use/). The GISAID data were used to define the proportion of travellers infected with circulating VOC and VOIs, for incoming travel to Canada, at the level of the country of departure. It was assumed the proportion of variants in the travellers were the same as reported in GISAID during a three-week period (which includes the week modelled and the two prior weeks). One limitation of this assumption pertains to sampling bias that might occur if sampling efforts were targeted – for example: sequencing people who had 1) recently travelled internationally and returned infected with SARS-CoV-2; 2) had close contact with someone returning infected from international travel; 3) visited or live in areas with unusual or unexpected increases in incidence of COVID-19 cases; and 4) become infected with SARS-CoV-2 after being recently vaccinated. We could not account for error from targeted sampling because the purpose of GISAID database was to report on the spatial distribution of sequenced SARS-CoV-2 variants and not provide information associated to the samples concerning surveillance objectives or individual travel history and exposure location. Another limitation of using the GISAID data was that the number of samples sequenced may have been insufficient to represent the national profile of variants. We could reduce error from low sampling effort by restricting VOC and VOI model outputs to countries with at least 20 sequenced samples. In summary, the model output estimates for VOC and VOI could still contain error from target sampling and low sampling effort.

| 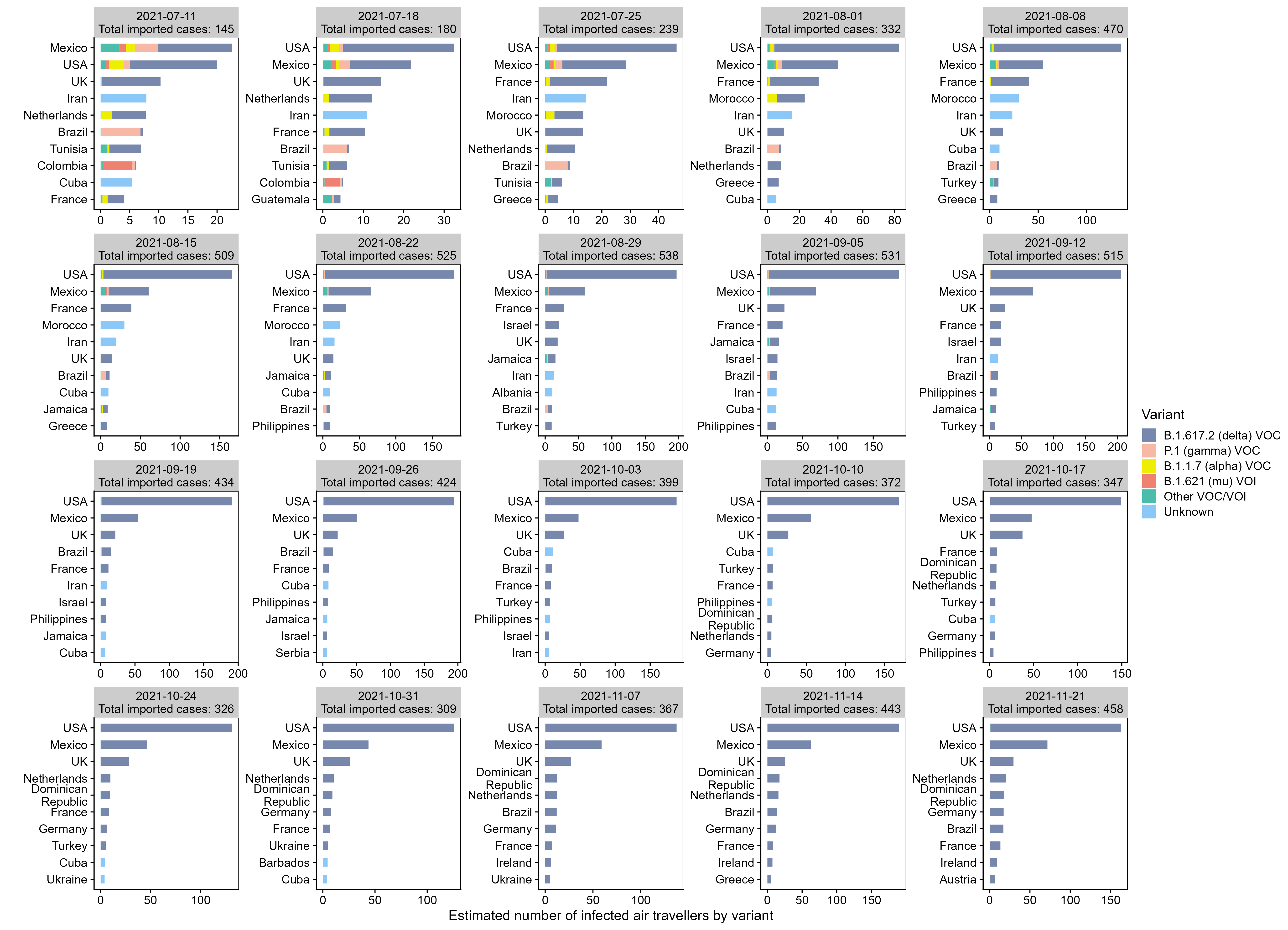 |
| --- |

**Figure A2:** Distribution of imported variants among the top ten countries most at risk of COVID-19 importation per week.

1. References

1. Zhao N, Liu Y, Smargiassi A, Bernatsky S. Tracking the origin of early COVID-19 cases in Canada. Int J Infect Dis. 2020;96:506-8.

2. Detsky AS, Bogoch, II. COVID-19 in Canada: Experience and response to waves 2 and 3. JAMA. 2021;326(12):1145-6.

3. Government of Canada. Timeline - CBSA border measures 2021. updated April 23, 2021. Available from: <https://www.publicsafety.gc.ca/cnt/trnsprnc/brfng-mtrls/prlmntry-bndrs/20210907/05-en.aspx>. Accessed: 2022-11-02

4. Transport Canada. Trasportation in Canada 2021; comprehensive report. 2021.

5. Government of Canada. Statement from the Chief Public Health Officer of Canada on January 7, 2021. Ottawa, ON, Canada.2021. Available from: <https://www.canada.ca/en/public-health/news/2021/01/statement-from-the-chief-public-health-officer-of-canada-on-january-7-2021.html>. Accessed: 2023-01-16

6. Government of Canada. Government of Canada will remove pre-entry test requirement for fully vaccinated travellers on April 1 2022. Available from: <https://www.canada.ca/en/public-health/news/2022/03/government-of-canada-will-remove-pre-entry-test-requirement-for-fully-vaccinated-travellers-on-april-1.html>. Accessed: 2023-01-16

7. Government of Canada. Travel Advisory: Reminder – On September 7, new measures for fully vaccinated international travellers to Canada will come into force 2021. Available from: <https://www.canada.ca/en/border-services-agency/news/2021/09/travel-advisory-reminder--on-september-7-new-measures-for-fully-vaccinated-international-travellers-to-canada-will-come-into-force.html>. Accessed: 2022-07-29

8. Government of Canada. Travellers’ testing, isolation and quarantine obligations. 2021. Available from: <https://www.publicsafety.gc.ca/cnt/trnsprnc/brfng-mtrls/prlmntry-bndrs/20210722/020/index-en.aspx>. Accessed: 2023-01-16

9. CBSA's Integrated Data Warehouse, with aggregated data being accessed through IBM COGNOS Analytics.

10. ArriveCAN. 2.21 ed: Government of Canada; 2021.

11. Canada Border Services Agency. Contact Trace. Version 3.9.2. 2021.

12. Johansson MA, Wolford H, Paul P, Diaz PS, Chen T-H, Brown CM, et al. Reducing travel-related SARS-CoV-2 transmission with layered mitigation measures: symptom monitoring, quarantine, and testing. BMC Med. 2021;19:1-13.

13. Zhen W, Smith E, Manji R, Schron D, Berry GJ. Clinical evaluation of three sample-to-answer platforms for detection of SARS-CoV-2. Journal of clinical microbiology. 2020;58(8):e00783-20.

14. Wu SL, Mertens AN, Crider YS, Nguyen A, Pokpongkiat NN, Djajadi S, et al. Substantial underestimation of SARS-CoV-2 infection in the United States. Nature communications. 2020;11(1):1-10.

15. Ritchie H, Mathieu E, Rodés-Guirao L, Appel C, Giattino C, Ortiz-Ospina E, et al. Coronavirus pandemic (COVID-19). Our World in Data. 2020.

16. Centers for Disease Control Prevention. COVID-19 Response. COVID-19 case surveillance public data access, summary, and limitations 2021. Available from: <https://www.cdc.gov/coronavirus/2019-ncov/cases-updates/cases-in-us.html>:. Accessed: 2021-09-29

17. JHU. Available from: <https://coronavirus.jhu.edu/vaccines/us-states>. Accessed: 2021-09-29

18. Open COVID-19 Data Working Group. Detailed epidemiological data from the COVID-19 outbreak 2020. Available from: <http://virological.org/t/epidemiological-data-from-the-ncov-2019-outbreak-early-descriptions-from-publicly-available-data/337>. . Accessed: 2021-09-29

19. World Health Organization. WHO COVID-19 detailed surveillance data dashboard. Available from: <https://app.powerbi.com/view?r=eyJrIjoiYWRiZWVkNWUtNmM0Ni00MDAwLTljYWMtN2EwNTM3YjQzYmRmIiwidCI6ImY2MTBjMGI3LWJkMjQtNGIzOS04MTBiLTNkYzI4MGFmYjU5MCIsImMiOjh9>. Accessed: 2021-09-29

20. Dong E, Du H, Gardner L. An interactive web-based dashboard to track COVID-19 in real time. The Lancet infectious diseases. 2020;20(5):533-4.

21. United States Census Bureau. Table 2. Resident population for the 50 states, the Distric of Columbia, and Puerto Rico: 2020 CENSUS. Available from: <https://www.census.gov/data/tables/2020/dec/2020-apportionment-data.html>. Accessed: 2023-02-01

22. The World Bank. Population, total - Tuvalu. Available from: <https://data.worldbank.org/indicator/SP.POP.TOTL?locations=TV>. Accessed: 2021-09-29

23. Jessica A. Bell , Nuzzo JB. Global health security index: advancing collective action and accountability amid global crisis 2021. Available from: <www.GHSIndex.org>. Accessed: 2023-09-06

24. The World Bank. GNI per capita, Atlas method (2019) 2019. Available from: <https://data.worldbank.org/indicator/NY.GNP.PCAP.CD>. Accessed: 2023-09-13

25. MRC Centre for Global Infectious Disease Analysis. Imperial College London. COVID-19 LMIC Reports 2020. Available from: <https://mrc-ide.github.io/global-lmic-reports/>. Accessed: 2022-11-02

26. Institute for Health Metrics and Evaluation (IHME). COVID-19 Projections. Seattle, USA: IHME, University of Washington 2022. Available from: <www.healthdata.org/covid/updates>. Accessed: 2022-11-02

27. Voysey M, Clemens SAC, Madhi SA, Weckx LY, Folegatti PM, Aley PK, et al. Safety and efficacy of the ChAdOx1 nCoV-19 vaccine (AZD1222) against SARS-CoV-2: an interim analysis of four randomised controlled trials in Brazil, South Africa, and the UK. The Lancet. 2021;397(10269):99-111.

28. Omrani AS, Tleyjeh IM. Which are the best coronavirus disease 2019 vaccines? Clinical Microbiology and Infection. 2021.

29. Lopez Bernal J, Andrews N, Gower C, Gallagher E, Simmons R, Thelwall S, et al. Effectiveness of Covid-19 vaccines against the B. 1.617. 2 (Delta) variant. N Engl J Med. 2021:585-94.

30. Sharma K, Koirala A, Nicolopoulos K, Chiu C, Wood N, Britton PN. Vaccines for COVID-19: where do we stand in 2021? Paediatr Respir Rev. 2021.

31. Pouwels KB, Pritchard E, Matthews PC, Stoesser N, Eyre DW, Vihta K-D, et al. Effect of Delta variant on viral burden and vaccine effectiveness against new SARS-CoV-2 infections in the UK. Nat Med. 2021:1-9.

32. Shu Y, McCauley J. GISAID: from vision to reality. Eurosurveillance. 2017;22(13).
